# Using the kalman filter with Arima for the COVID-19 pandemic dataset of Pakistan

**DOI:** 10.1016/j.dib.2020.105854

**Published:** 2020-06-12

**Authors:** Muhammad Aslam

**Affiliations:** Department of Statistics, Bahauddin Zakariya University, Multan 60800, Pakistan

**Keywords:** Arima model, COVID-2019 pandemic, Forecast, Holt-winters’ method, Infection control, Kalman filter, SutteARIMA, State space model

## Abstract

The current pandemic of the Novel Corona virus (COVID-19) has resulted in multifold challenges related to health, economy, and society, etc. for the entire world. Many mathematical epidemiological models have been tried for the available data of the COVID-19 pandemic with the core objective to observe the trend and trajectories of infected cases, recoveries, and deaths, etc. However, these models have their own assumptions and parameters and vary with regional demography. This article suggests the use of a more pragmatic approach of the Kalman filter with the Autoregressive Integrated Moving Average (ARIMA) models in order to obtain more precise forecasts for the figures of prevalence, active cases, recoveries, and deaths related to the COVID-19 outbreak in Pakistan.

**Specifications Table**

**Table:** 

**Subject**	Infectious diseases
**Specific subject area**	Time-series and econometric modeling
**Type of data**	Table Graph
**How data were acquired**	The data were acquired from the official website maintained by the Government of Pakistan (http://covid.gov.pk/). Instruments: Programming language R and its packages, “TSPred” “forecast”, and “SutteForecastR” were used for the analyses.
**Data format**	The data are in raw format and have been analyzed. An Excel file with data has been uploaded.
**Parameters for data collection**	The dataset consists of daily reported total (cumulative) confirmed & active cases of COVID-19, recoveries, and deaths. The parameters were used for the Kalman-filtered ARIMA models.
**Description of data collection**	The daily prevalence data of cumulative confirmed COVID-19 cases, active cases, recoveries, and deaths in Pakistan from February 26, 2020, to April 30, 2020, were collected from the official website of the Government of Pakistan (http://covid.gov.pk/), and MS-Excel 2019 was used to build a time-series database for further analysis.
**Data source location**	Ministry of National Health Services Islamabad Pakistan
**Data accessibility**	Raw data can be retrieved from http://covid.gov.pk/stats/pakistan

**Value of the Data**

These data are useful because they provide a forecast for not only the number of confirmed cases reflecting the outbreak of the COVID-2019 pandemic but the number of active cases, recoveries, and deaths as well, thus representing a valid and objective tool for monitoring infection prevalence and control.
The institutions involved in the command and control of the pandemic and the general public can both benefit from these data when using outcomes of the fitted models.
With the help of fitted models and the use of available data, some reliable forecasts of infected cases, active cases, recoveries, and deaths can be made for the future.
Better measures about epidemic management can be taken after applications of the fitted Kalman-filtered ARIMA models. The health practices, facilities, need and intensity of lockdown, and efficient quarantining period can be assessed.

## 1 Data description

**Fig. 1:**
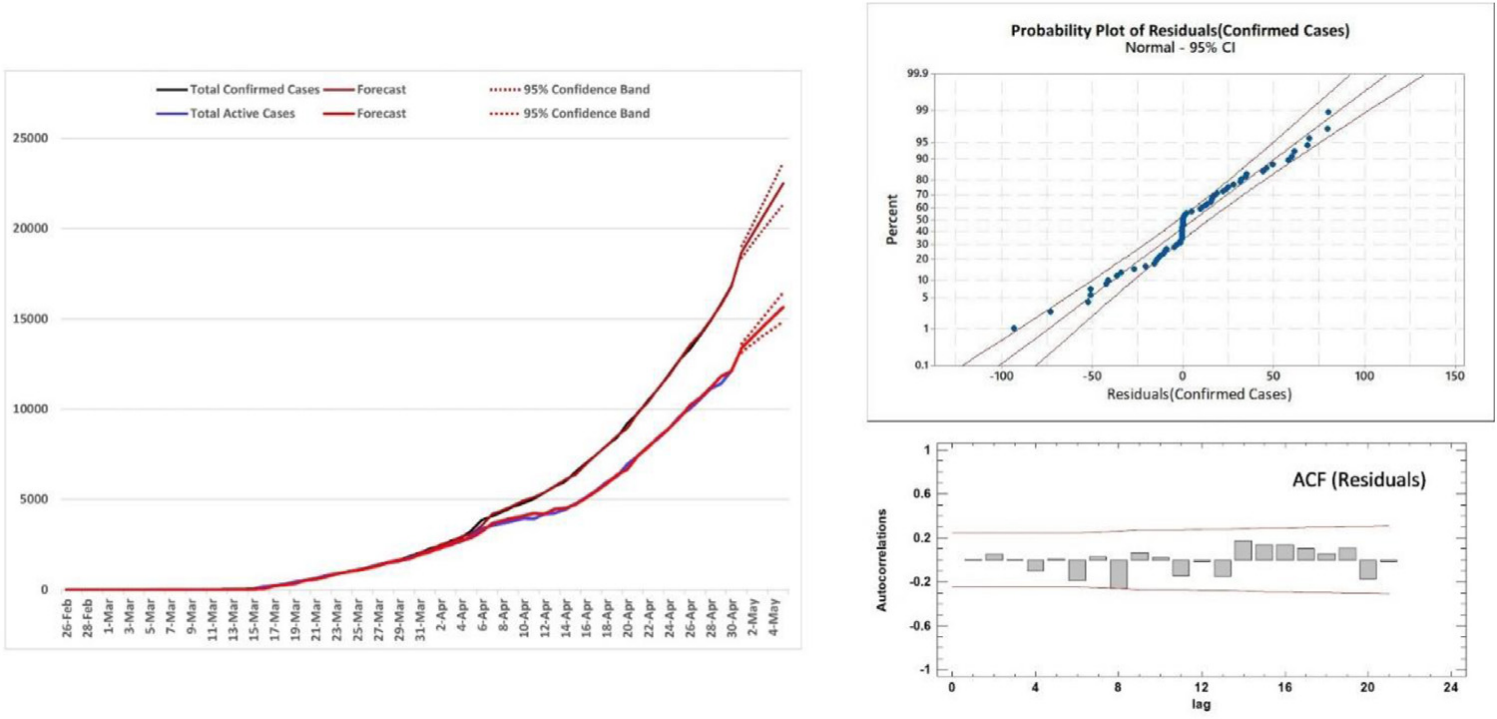
Forecast of the cumulative confirmed and active cases of COVID-19 in Pakistan.

**Fig. 2:**
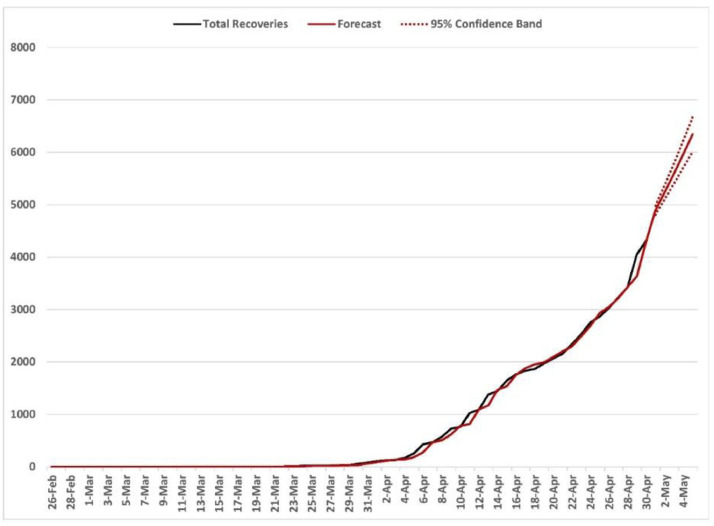
Forecast of the total recoveries from COVID-19 in Pakistan.

**Fig. 3:**
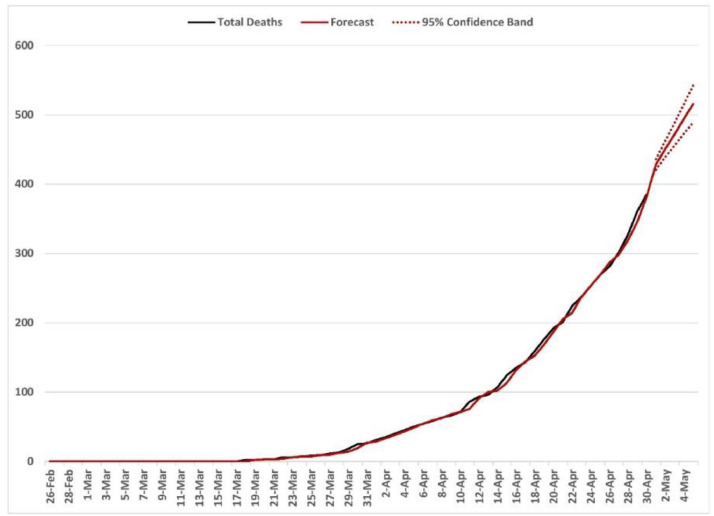
Forecast of the total deaths due to COVID-19 in Pakistan.

**Fig. 4:**
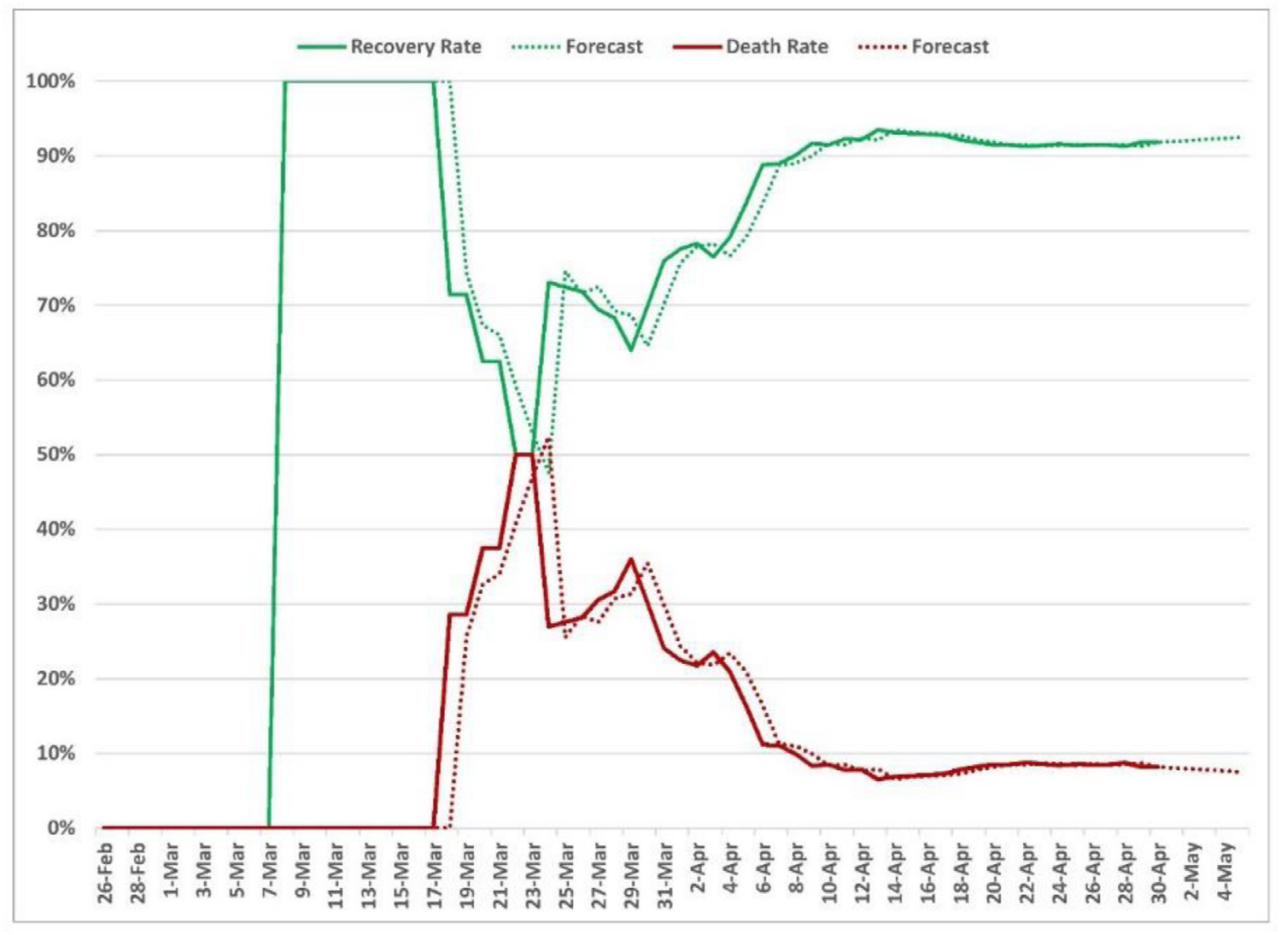
Forecast of the recovery-rate & death-rate as outcomes of the closed cases (total recovered & deceased).

The daily prevalence data (number of cumulative confirmed cases) of COVID-19, number of total active cases, recoveries, and deaths from Pakistan for 65 days from February 26 to April 30, 2020 were collected from the official website of the Government of Pakistan (http://covid.gov.pk/) [1]. The obtained raw data were tabled in MS-Excel 2019 to build a time-series database for further use. The Kalman filters with the ARIMA models were applied to the dataset, in relation to the COVID-19 pandemic. Fig. 1 shows that the overall prevalence and active cases of COVID-19 have an increasing trend (in the form of exponential curves) depicting an epidemic in Pakistan. Fitting of normal distribution to the residuals and autocovariance function (ACF) were also displayed in Fig. 1. The daily situation of total (cumulative) recoveries and deaths with a five-day-ahead forecast has been displayed in Fig. 2 & 3, respectively. The recovery- & death-rates (out of closed cases) are presented in Fig. 4. Table 1 compares different fitted models showing the results of fitting data (April 26–30, 2020) of total confirmed cases. Table 2 reports the figures about forecast of prevalence, active cases, recoveries, and deaths with relative 95% confidence intervals.

**Table 1:** Comparison: The results of fitting data (April 26–30, 2020) of total confirmed cases of COVID-19 in Pakistan.

Date	Actual reported cases	Holt-winters’ method (APE)	SutteARIMA (APE)	KF-ARIMA (APE)
26-Apr-20	13,328	13,171 (0.0118)	13,483 (0.0116)	13,535 (0.0155)
27-Apr-20	14,079	13,842 (0.0168)	14,174 (0.0068)	14,152 (0.0052)
28-Apr-20	14,885	14,513 (0.0250)	14,812 (0.0049)	14,937 (0.0035)
29-Apr-20	15,827	15,184 (0.0406)	15,625 (0.0128)	15,783 (0.0028)
30-Apr-20	16,817	15,855 (0.0572)	16,684 (0.0079)	16,782 (0.0021)
	MAPE	0.0303	0.0088	**0.0058**

**Table 2:** Forecast values for the five days after the analysis for the prevalence, active cases, recoveries, and deaths related to COVID-19 in Pakistan.

Date	Prevalence (95% confidence interval)	Total active cases (95% confidence interval)	Total recoveries (95% confidence interval)	Total deaths (95% confidence interval)
01-May-20	18,709 (18,377 –19,042)	13,386 (13,148 – 13,624)	4895 (4808 – 4982)	428 (421 – 436)
02-May-20	19,659 (19,148 – 20,169)	13,966 (13,603 – 14,329)	5242 (5106 – 5378)	451 (439 – 462)
03-May-20	20,609 (19,900 – 21,318)	14,538 (14,037 – 15,038)	5599 (5406 – 5792)	472 (456 – 488)
04-May-20	21,559 (20,630 – 22,488)	15,099 (14,448 – 15,750)	5966 (5709 – 6223)	494 (473 – 515)
05-May-20	22,509 (21,341 – 23,677)	15,652 (14,840 – 16,464)	6342 (6013 – 6671)	516 (489 – 542)

## 2 Experimental design, materials, and methods

The primary purpose of using time-series models, such as autoregressive moving average (ARMA) models, is to forecast. These models are applicable to stationary (stable) time-series, and the Augmented Dickey-Fuller (ADF) unit-root test [2] is commonly used to check whether the time-series is stationary or not. In practice, log transformation and differences are the preferred approaches to stabilize the time-series [3]. Seasonal and non-seasonal differences are used to stabilize the term trend and periodicity [4]. If a time-series becomes stationary after differencing, then an ARMA model used for that transformed series is referred to as an ARIMA model where “I (integrated)” reflects the order of differencing. Usually, the Box-Jenkins methodology [3] is used to fit an appropriate ARIMA model.

There is another class of models called the state space models (SSMs). An SSM involves dynamics for an unobserved stochastic process called the state and a distribution for the actual observations called function of the state. Every ARIMA model is actually an SSM. The Kalman filter (KF) is an algorithm that works in the context of an SSM to compute the sequence of filtering distribution (the distribution of the current state) and compute the likelihood of the data. Thus, the use of the KF with ARIMA could provide better predictions and forecasts through optimal estimates. However, the presence of outliers may affect the optimality while using the KF [5,6].

A package “TSPred” of the R language [7] was used for the KF algorithm with the most appropriate ARIMA model for the data about COVID-19. After applying the ADF test, the data-series of cumulative confirmed cases was made stationary with the first difference and the most suitable order of ARIMA, following the Box-Jenkins methodology [3], was ARIMA (1,1,1). Similar routines were followed for the other data series.

The proposed method of forecasting was compared with one of the classical methods (i.e., the Holt-Winters method [8]) and one of the latest available models i.e., the SutteARIMA [9,10]. For the evaluation of the forecasting methods, many popular measures for the accuracy of forecasting were considered. However, we report here the absolute percentage error (APE) and mean APE (MAPE).

$$APE_{t}=\left|\frac{Y_{t}-F_{t}}{Y_{t}}\right|,$$

$$MAPE=\sum_{t=1}^{T}APE_{t},$$

where *Y_t_* and *F_t_* are observed and forecast values, respectively at time *t*.

Four previous studies were considered as reference for the methodology of the analysis [6,[10], [11], [12].

To determine the prevalence (cumulative confirmed cases) of COVID-19 and active cases in Pakistan, the KF algorithm was used for the best-fitted ARIMA models, following the available methodology [6]. Following Benvenuto et al. [8], logarithmic transformation was performed to evaluate the influence of seasonality on the forecast.

As recoveries from the infection of COVID-19 and deaths due to COVID-19 depend on the prevalence of the disease, it is not appropriate to fit some direct models to the data of recoveries and deaths. Therefore, it is proposed to model the ratios (*R_t_*/*C_t_* and *D_t_*/*C_t_*) where *C_t_* = cumulative confirmed cases; *R_t_* = cumulative recoveries; and *D_t_* = cumulative deaths at time *t*. The forecasts of these ratios can easily be converted into the respective figures of total recoveries and deaths after considering the results of the model for confirmed cases.

The forecasting accuracy was compared for three methods used–the Holt-Winters method, SutteARIMA, and KF-ARIMA–for all the data-series. We report here the results for the cumulative confirmed cases of COVID-19 for five days (April 26–30, 2020). The actual reported values, forecast values and APE and MAPE are displayed in Table 1 for the Holt-Winters method (α=0.8301,β=0.6144), SutteARIMA, and KF-ARIMA (1,1,1). The lowest MAPE (0.0058) advocates the choice of the proposed KF-ARIMA.

The forecast figures were computed for the next five days (i.e., May 1 to May 5, 2020) of the used dataset. These figures with relative 95% confidence intervals are reported in Table 2. Although the spread of the virus seems to be increasing slightly, the number of recoveries are also increasing with a relative small number of deaths.

Finally, it may be noted that forecasting is always a tricky subject and there can be a number of candidate models that can be fitted to the available data. However, any fitted model may not be fully accurate due to the complex, evolving, and varying environmental, social, and economic conditions of different countries. Thus, predictions and forecasts are uncertain by nature. The above presented models and forecasts do not consider the regional demography, and the actual figures may change due to many administrative measures like intensity of lockdown, policy of quarantining and health facilities etc. Thus, readers should be careful while interpreting these forecasts.

## Declaration of Competing Interest

The author declares no known competing financial interests or personal relationships that could have appeared to influence the work reported in this paper.

## References

[1] Ministry of National Health Services, Islamabad, Pakistan, 2020. http://covid.gov.pk/stats/pakistan.

[2] Cheung Y.W., Lai K.S. (1995). Lag order and critical values of the augmented Dickey–Fuller test. J. Bus. Econ. Stat..

[3] Box G.E., Jenkins G.M., Reinsel G.C., Ljung G.M. (2015). Time Series Analysis: Forecasting and Control.

[4] Chatfield C., Prothero D.L. (1973). Box‐Jenkins seasonal forecasting: problems in a case‐study. J. Royal Stat. Soc. Ser. A (General).

[5] Kalman R.E. (1960). A new approach to linear filtering and prediction problems. J. Basic Eng..

[6] Sholl P., Wolfe R.K. (1985). The Kalman filter as an adaptive forecasting procedure for use with Box-Jenkins ARIMA models. Comput. Ind. Eng..

[7] https://cran.r-project.org/web/packages/TSPred/TSPred.pdf, 2018.

[8] Goodwin P. (2010). The holt-winters approach to exponential smoothing: 50 years old and going strong. Foresight.

[9] Ahmar A.S. (2018). A comparison of α-Sutte Indicator and ARIMA methods in renewable energy forecasting in Indonesia. Int. J. Eng. Technol..

[10] Ahmar A.S., del Val E.B. (2020). SutteARIMA: short-term forecasting method, a case: COVID-19 and stock market in Spain. Sci. Total Environ..

[11] Domenico B., Giovanetti M., Lazzaro V., Silvia A., Massimo C. (2020). Application of the ARIMA model on the COVID-2019 epidemic dataset. Data Brief.

[12] Wang Y.W., Shen Z.Z., Jiang Y. (2018). Comparison of ARIMA and GM(1,1) models for prediction of hepatitis B in China. PLoS ONE.

